# Immunogenicity evaluation of the HIV-1 Tat containing polyepitope DNA vaccine adjuvanted with CpG-ODNs in mice

**DOI:** 10.22038/ijbms.2021.52890.11923

**Published:** 2021-03

**Authors:** Ehsan Ollah Jazaeri, Atiyeh Mahdavi, Asghar Abdoli

**Affiliations:** 1 Department of Biological Sciences, Institute for Advanced Studies in Basic Sciences (IASBS), Zanjan, Iran; 2 Department of Hepatitis and AIDS, Pasteur Institute of Iran, Tehran, Iran

**Keywords:** Cellular immunity, CpG oligodeoxynucleotides, DNA vaccine, HIV-1, Humoral immunity

## Abstract

**Objective(s):**

HIV-1 is still considered a serious threat to human health, and accessibility of a suitable and efficient vaccine is urgently needed to address the disease burden. DNA vaccines employ the cells of the vaccinated hosts for *in situ* production of the vaccines. This strategy is an alternative and effective approach for traditional vaccination against high-risk pathogens, e.g., HIV-1. On the other hand, polyepitope vaccines, containing several immunogenic and conserved epitopes from virus vital regulatory and structural proteins, could more efficiently induce cellular and humoral immune responses against different clades of the virus.

**Materials and Methods:**

Herein, we comparatively investigated the immunogenic potency of the HIV-1 polytope DNA vaccine containing CpG oligodeoxynucleotides (CpG-ODNs) in BALB/c mice. To this end, after verifying the expression of the recombinant sequence in the eukaryotic HEK 293 cell line, it was amplified and extracted in the prokaryotic host cells (*E. coli* DH5α)) and then formulated and administered intramuscularly (IM) to the experimental mice (on days 0, 14, and 28) with and without CpG-ODNs adjuvant.

**Results:**

Taken together, the results demonstrated that CpG-ODNs adjuvanted DNA vaccine could significantly elicit cellular and humoral immune responses in the immunized animals in comparison with the control ones (*P*<0.05).

**Conclusion:**

Regarding the obtained results and also considering the advantages of polytopic and DNA vaccines, this approach might be considered a new regimen in HIV-1/AIDS vaccination.

## Introduction

Since identification of HIV-1/AIDS, many people have been affected by the virus worldwide and almost 50% of them have died. Thus, accessibility to a suitable efficient vaccine is in urgent need to address the disease burden (1). Although different types of vaccines have been developed (2), they failed to provide the desired protection due to some stumbling blocks, including the high genetic diversity of the virus, its capacity to evade the adaptive immune response, induction of weak antibody responses, etc. (3). 

 Discovery of DNA vaccines (1980s) circumvented the need for production of protein purification vaccines in laboratories. These vaccines employ the cells of the vaccinated hosts for *in situ* production of antigens and keep long-lived effector activity (2, 4, 5). In this vaccination approach, the DNA is finally converted to the antigen of interest following the plasmid delivery and can then induce immune responses and mimics the natural infection. This *in situ* intracellular production of antigens not only elicits humoral immunity but also leads to induction of cellular immune responses (2, 4, 5). It is established that an efficient HIV-1 vaccine has to induce durable cross-clade humoral and cellular immune responses (1). To meet these critical requirements, various attempts have been made to assemble cocktails of conserved and protective immunogenic epitopes derived from different HIV-1 proteins. This approach has some valuable advantages including no risk for integration of gene into the host cell, no infection triggering during immunization, flexibility in polyepitope DNA vaccine designing, etc. The latter can elicit several types of immune responses that broaden the spectrum of induced immune responses and induce robust immunogenicity of vaccines (6). Designing an effective polyepitope vaccine needs some important considerations such as the immunogenicity of the desired epitope, conservancy of the desired epitope in different HIV-1 clades, and similar affinity of the selected epitopes for binding to MHC molecules (7).

Several clinical trials have shown that DNA vaccines induce relatively modest immunogenicity in large animals, especially humans (8). Therefore, the immunogenicity of DNA vaccines could be reinforced by using different vectors, adjuvants, or delivery systems (4). 

In this research, the immunogenicity of the HIV-1 ployepitopic DNA vaccine in mice was evaluated in the presence and absence of CpG-ODNs as an adjuvant. In this regard, after verification of the recombinant vaccine expression in the eukaryotic host cells (HEK 293 cell line), its amplification (in *E*. *coli* DH5α) and extraction was carried out and it was then formulated either alone or adjuvanted with CpG-ODNs and finally administered IM to the experimental mice on days 0, 14, and 28. The immunogenicity of the candidate vaccines was then comparatively assessed and analyzed.

## Materials and methods

***Materials***

Goat anti-mouse (IgG_1_ and IgG_2a_) secondary antibodies and CpG oligodeoxynucleotides (CpG-ODNs) adjuvant were purchased from Sigma (St. Louis, MO, USA). Ampicillin antibiotic was provided from Invitrogen (Carlsbad, CA, USA). The kits for the 5-Bromo-2-deoxyuridine (BrdU) test and lactate dehydrogenase (LDH) assay were provided by Takara. The restriction endonucleases were purchased from Fermentas. Cell culture medium RPMI-1640 was supplied by Gibco. Cytokine assay kit was obtained from Due Set, and Merck provided all of the other chemicals. Data reproducibility was confirmed by at least three independent experimental repeats and the presented results are typical experimental data.

***Polyepitope vaccine characteristics***

The pcDNA polyepitope vector was obtained from Iran’s Pasteur Institute (IPI, Tehran, Iran). Some important criteria have been carefully met in designing the recombinant vector. It includes six amino acid fragments from the immunogenic and conserved epitopes of the HIV-1 Tat, Pol, Gag, and Env antigens that have been tandemly conjoined as a full-length polytope using the LosAlamos HIV immunology database (http://www.hiv.lanl.gov/content/immunology/index.html). 3 spacer sequences (AAA and AAY) have been inserted between the epitopes for optimization of cleavage by the proteasome and also reducing the possibility of formation of junctional epitopes. CLC Main Workbench 5.5 software (CLC bio, USA) was applied to assess and reduce the hydrophobic residue accumulation (9) (supplementary table).

***Western blotting analysis***

After ensuring the presence of the recombinant sequence in the pcDNA3.1 vector (using double digestion with *Bam*HI and *Xho*I restriction endonucleases) and its transfection into the eukaryotic host cell (HEK 293 cell line, as one of the potent eukaryotic expression systems), the expression of the candidate vaccine was verified by using Western blot analysis. Briefly, HEK 293 cell line was seeded in a 75 cm^2^ Cell Culture Flask and once the confluency of the cells reached 70–90 percent, they were transfected by the recombinant pcDNA3.1 expression vector, using lipofectamine 2000 reagent. 

48 and 72 hr post transfection, cell harvesting was carried out by centrifugation and the cells were then lysed in a lysis buffer (40 mM Tris HCl (pH 7.4), 2 M BioLite, 7 M urea, 50 mM dithiothreitol (DTT), 0.2% 3-[(3- Cholamidopropyl)dimethylammonio]-1-propanesulfonate (CHAPS), 4% (w/v) thiourea, protease inhibitor cocktail (Roche) and 200 µM phenylmethylsulfonyl fluoride (PMSF). We did not determine the cell lysate concentration (total protein), and to qualitatively confirm the protein expression, we loaded and ran the maximum possible amount of the extract on 12% sodium dodecyl sulfate-polyacrylamide gel electrophoresis (SDS-PAGE) well. Blotting was then carried out according to the procedure (10), and a chemiluminescence image station (Kodak) was used for visualization of the protein band. 

***Amplification and expression of the recombinant vector***

The recombinant pCDNA3.1 vector was extracted and transformed into the competent cells (*E*. *coli* (DH5α)) by a chemical method (11). The transformed cells were cultivated for 12–16 hr in 250 ml of Luria-Bertani broth (LB) medium containing ampicillin (50 μg ml^−1^) in 1 L Erlenmeyer and the temperature and vigorous shaking were adjusted to 37 °C and 250 rpm, respectively. Harvesting of the cells was carried out by centrifugation at 5000 g, 4 °C, 20 min (11). After removing the supernatant, the pellet was used for plasmid extraction using a commercial endotoxin-free Qiagen plasmid extraction kit. The content of endotoxin was < 0.1 EU/µg DNA (endotoxin units per µg plasmid DNA), and the amount of the supercoiled plasmid was > 80%.

***Mice and vaccination protocol***

The immunogenicity of the candidate DNA vaccines (with and without CpG-ODNs adjuvant) was tested in 6–8 week old BALB/c mice. National Institutes of Health Guidelines for the Care and Use of Laboratory Animals (NIH publications No. 8023, revised 1978) were carefully considered during the work. The animals were divided randomly into 4 groups and 6 mice per group. Immunization was carried out intramuscularly (IM) 3 times at 2-week intervals (on days 0, 14, and 28) with the DNA vaccine alone or adjuvanted with CpG-ODNs (Table 1).

***Measurement of antibody levels***

Bleeding was done seven days after the final vaccination. Sera samples from the immunized mice were separated and our previously optimized indirect ELISA method was used to measure the levels of IgG subclasses (IgG_1_ and IgG_2a_) (7). 

In brief, 100 μl of 10 μg/ml of the protein vaccine buffered with PBS was coated into 96-well ELISA Maxisorp plates overnight and the temperature was adjusted to 4 °C. The protein was recombinantly expressed in a prokaryotic system (*E*. *coli* BL21 (DE3)) and purification was performed by Ni-NTA affinity resin. The levels of the elicited antibodies were finally determined according to our previous method using horseradish peroxidase (HRP) conjugated goat anti-mouse IgGs (7).

***Cellular immunity assays***

For assessment, the cellular immune responses induced by the HIV-1 polyepitope DNA vaccines, proliferation of lymphocytes, production of interferon-gamma (IFN-γ), and cytotoxic activity of T lymphocytes (CTL) were measured as the hallmarks of cellular immunity. For this purpose, 2 weeks after the third and last vaccination, isolation of the vaccinated mice splenocytes, preparation of single-cell suspensions, and development of ELISA assays were performed as mentioned previously (7). To assay the lymphocyte proliferation which was performed using 5-Bromo-2-deoxyuridine (BrdU) kit, concanavalin-A (Gibco, with a final concentration 5 μg ml^-1^) was utilized as the positive control while complete culture medium and unstimulated wells were respectively used as the blank and negative control, Stimulation of the cells’ suspensions was carried out using the HIV-1 corresponding polyepitope protein as antigen recall. The optical density (OD) values were recorded at 450 nm with a Microplate Reader (Biohit 8000, USA), and the stimulation index (SI) calculated as OD of stimulated wells/OD of unstimulated wells, were used to report the cell proliferation. 

Measurement of IFN-γ produced by the immunized mice was also carried out using R&D Systems commercial kit and IFN-γ cytokine quantity (pg ml^-1^) was calculated by plotting the standard curve. To assay the CTL activity, the suspensions obtained from splenocytes of the vaccinated animals were used as effector cells and the mouse myeloma Sp2-0 tumor cell line was applied as the target cells. The standard lactate dehydrogenase (LDH) assay was performed for *in vitro* detection of the cell-mediated killing of target cells at various ratios of effector/target cells. The percent of cytotoxicity was finally accounted by the corresponding formula (7). 

The complete cell lysate was obtained using Triton X-100 (1%) and the spontaneous release was examined with target cells prepared in RPMI-1640 containing BSA (2%).

***Statistical analysis***

All experiments presented in this paper were performed in triplicates and repeated 3 times. Procession and analysis of data were carries out using Graphpad Prism software (version 6.01) and presented as means±standard deviation (SD) using the one-way ANOVA method. 

## Results

***The polyepitope DNA vaccine production and preparation***

**Figure 1:**
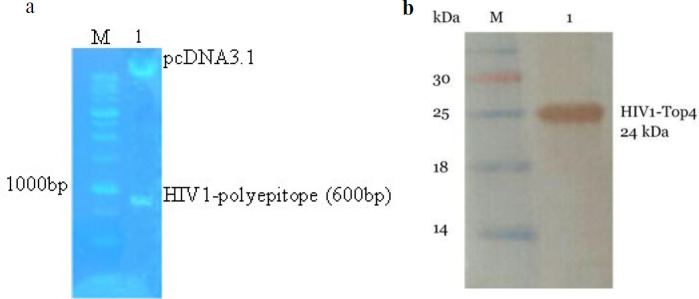
(a) Analysis of the extracted pcDNA3.1-HIV-1-polyepitope vector on 1% agarose gel. Lane 1, the pcDNA3.1-HIV-1- polyepitope vector that was digested by BamHI and XhoI. The 600 bp bond shows the HIV-1-polyepitope fragment; lane M, molecular size marker. (b) Western blotting of the pure recombinant protein; lane M, molecular size marker; lane 1, the pure HIV-1-polyepitope recombinant protein expressed in the eukaryotic host cell (HEK 293)

The existance of the HIV-1 polyepitope DNA fragment in the recombinant pcDNA3.1 plasmid was verified using double enzymatic digestion and agarose gel analysis (1% agarose) (Figure 1a). The expression of the HIV-1 polyepitope protein in the HEK 293 cell line was also verified by Western blot analysis (Figure 1b). The vector amplification and extraction were then carried out and DNA concentration was determined using a spectrophotometer (Picodrop-Pico200, UK)

***Evaluation of humoral immunity***

**Figure 2:**
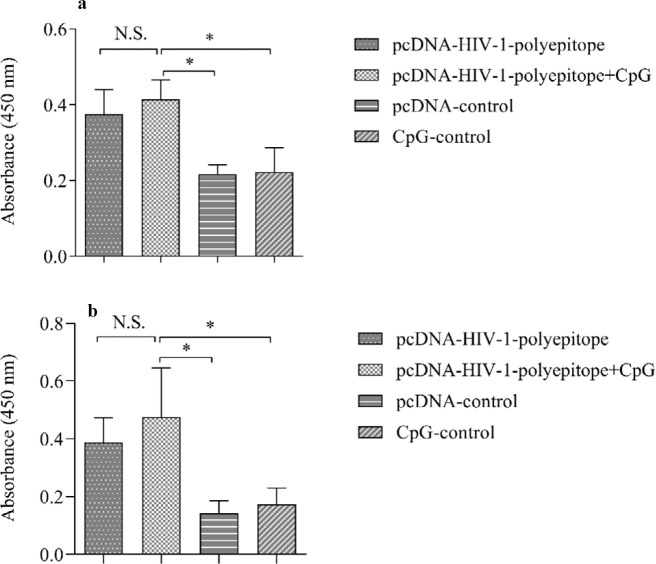
Examination of the humoral immunity of HIV-1 polytopic adjuvanted and non-adjuvanted DNA vaccines in mice. The test was carried out 1 week after the final administration and the titers of (a) IgG1 and (b) IgG2a were measured by an indirect ELISA method

The results of the assessment of the specific IgG_1_ levels in the serum samples of vaccinated animals suggested that immunization of the mice with the candidate DNA vaccines could significantly increase the antibody levels in the vaccinated animals in comparison with the control groups (Figure 2a). Similar results were also obtained for the specific IgG_2a_ amounts in the sera of the vaccinated mice (Figure 2b) (*P*<0.05). The animals vaccinated with the CpG-ODNs containing DNA vaccine produced greater amounts of both antibodies (IgG_1_ and IgG_2a_) in comparison with those who received the candidate DNA vaccine alone. Nevertheless, no significant difference was observed (*P*>0.05).

***Examination of cellular immunity***

**Figure 3:**
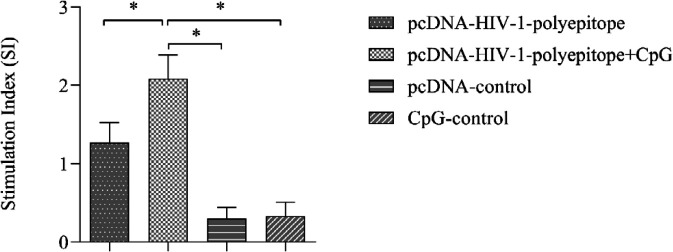
Proliferation of lymphocytes in the vaccinated mice. Administration of the candidate DNA vaccines was intramuscularly performed 3 times at 2-week intervals. Spleen lymphocytes were extracted from individual mice 14 days after the third and final immunization, cultured and stimulated with the HIV-1-polyepitope protein. The subsequent proliferation responses were analyzed and represented as the stimulation index (SI) values. The data are from triplicate experiments and expressed as the mean of SI values±SD of (*P*<0.05)

BrdU method was used for evaluation of the induction of lymphocyte proliferative responses and the amounts of SI values were calculated. Data showed that vaccination of animals with HIV-1 polyepitope DNA vaccines (with and without the CpG-ODNs adjuvant) could elicit proliferation of lymphocytes in the immunized mice in comparison with the control ones (Figure 3) and the difference was remarkable (*P*<0.05). 

**Figure 4:**
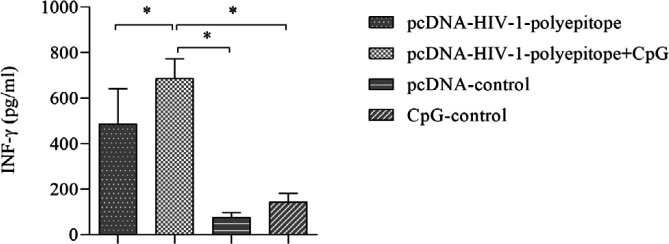
Assessment of cellular responses to the candidate DNA vaccines. Production of IFN-γ in the splenocytes isolated from the animals vaccinated with the polyepitopic DNA vaccines (in the presence and absence of CpG-ODNs) was evaluated using the ELISA method (*P*<0.05)

For further investigation of the cellular immune responses induced by the DNA vaccines, the amount of IFN-γ secreted by Th1 cells was evaluated using an ELISA method. IFN-γ secretion occurred upon specific recognition of the HIV-1 polyepitope antigens that had been expressed in the eukaryotic host cells and used as antigen recall. According to the results (Figure 4), increased secretion of IFN-γ was measured in response to the DNA candidate vaccines (*P*<0.05) and its quantity was remarkably higher in the animals receiving DNA vaccine adjuvanted with CpG-ODNs than in the group immunized with non-adjuvanted DNA vaccine, indicating elicitation of potent Th1-type responses in this group.

**Figure 5:**
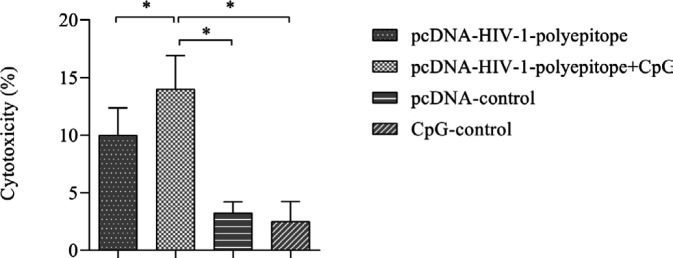
Induction of cytotoxicity responses against the HIV-1-polyepitope DNA vaccine was evaluated by lactate dehydrogenase assay. Data was indicated by the measurement of the amount of the LDH release as a result of the specific cytolysis of the cells that present the desired epitopes. Triton X-100 cell lysate was used as a positive control (*P*<0.05)

With regard to the determinant role of CTLs in the clearance of several viral infections, LDH was evaluated as one of the most important indicators of CTLs activity upon particular recognition of the antigens presented by APC cells. According to the results, the HIV-1 polyepitope DNA vaccine adjuvanted with CpG-ODNs could considerably increase the cytotoxicity responses in comparison with other formulations in the immunized animals (Figure 5). 

**Table 1:** The immunization protocol for the HIV-1 DNA vaccine candidates

**Group**	**Immunogen**	**Adjuvant**
pcDNA-HIV-1 polyepitope pcDNA-HIV-1 polyepitope +CpG-ODNs pcDNA-control CpG-ODNs-control	pcDNA-HIV-1 polyepitope vector (50 μg/1dose) pcDNA-HIV-1 polyepitope vector (50 μg/1dose) pcDNA3.1 vector (50 μg/1dose) CpG-ODNs (10 μg/1dose)	---- CpG-ODNs (10 μg/1dose) ---- ----

N.S.: Not Significant

## Discussion

HIV-1 is a significant challenge in terms of vaccine development. Traditional vaccines like live virus or even attenuated virus are very effective, although they are too risky against HIV-1. Therefore, other alternative vaccination modalities such as recombinant DNA or protein vaccines should be developed (5). In recent years, DNA based vaccine modality has been largely considered due to some brilliant privileges including safety, relative simplicity for construction, production and administration, scalability with high purity and stability (2, 12), and up to now, some researchers have applied naked DNA as a single vaccine modality. Several pieces of evidence show DNA vaccination has successfully protected macaques from chronic viremia (4). It is indicated that an efficacious protective vaccine against HIV-1 must provoke both protective antibodies and strong T cell responses. To this goal, neutralizing epitopes or conserved T cell epitopes have been utilized for HIV-1 polyepitope vaccine design. Taken together, this strategy has been promising in recent years for designing and producing new HIV-1 vaccines (13, 14).

Yang *et al*. employed some conserved epitopes as an HIV-1 DNA vaccine and their results suggested that the candidate vaccine could induce vigorous cellular immune responses and yielded partial protection (15). Reguzova and colleagues also reported expression of the HIV-1 polyepitope T cell immunogens, which placed together as a DNA vaccine construct, could cause CD8^+^ and CD4^+^ T cell responses in the immunized animals (16). The results of previous studies also revealed that the combination of genes encoding the important HIV-1 epitopes (from both structural and regulatory viral proteins) induced much more immunogenicity in comparison with the very same structural or regulatory genes when used alone (9, 17). So far, various regulatory and structural proteins of HIV-1, e.g., Pol, Gag, Vif, Nef, Tat, and Env have been considered to create polyepitope vaccines (18). These proteins are heavily involved in the life cycle and pathogenesis of the virus viral and are considered the best candidates to produce efficient and safe HIV/ADIS vaccines (7, 19). According to the literature, the inhibition of these viral vital proteins could noticeably cause the decline of the virus infectivity at different steps of disease (7, 9). Very effective control of the progression of AIDS is attainable using the HIV-1 Gag protein (20), and targeting the viral Pol and Tat proteins could greatly decrease the pathogenicity of the virus (9, 21). Considering the critical role of the HIV-1 Env protein in the virus-lymphocytes (T cells) interactions, the induction of immune responses against this viral protein could therefore affect the HIV-1 neutralization (9). 

Different researchers have also considered using effective adjuvants and different vaccine delivery systems in vaccine design. These approaches help significantly improve the efficiency of vaccines via modulation and enhancement of the immune responses and reduction of antigen doses (22). It is indicated that using efficient adjuvants in the HIV-1 candidate vaccine formulation would be a suitable approach to increase the efficacy of vaccines, and it elicited robust immune responses in vaccinated models (23). 

CpG-ODNs exist at high repetition in prokaryotic DNAs but rare in eukaryotic ones that are able to bind to their cognate TLR-9 receptors on surfaces of cells and cause the activation of the innate immune system. The innate immune activation leads to production of Th1 and pro-inflammatory cytokines and chemokines including IL-12, IL-6, IL-1, and TNF-α (2, 12, 24-28). By using CpG-ODNs as adjuvant in a vaccine formulation we could imitate the immune stimulatory activity of bacterial DNA when it infects eukaryotic cells. After internalization by target cells, CpG-ODNs reach the late endosomal/lysosomal compartment and are released to trigger immune stimulation by signaling cascades involving the up-regulation of NF-ĸB. This human-compatible adjuvant also improves the antigen-presenting function of macrophages, dendritic cells, and monocytes, induces the proliferation of B cells and indirectly stimulates the immunoprotective activity of natural killer cells when it recruits T cells to the site of CpG-ODNs administration (2, 12, 24-28). As a result, CpG-ODNs will be able to act as a potent vaccine adjuvant. Besides that, it could induce faster, longer-lasting, and stronger cellular and humoral immune responses which can be seen following either systemic or mucosal candidate vaccine administration (2, 12, 24-28). 

A study documented the ability of CpG-ODNs adjuvant in eliciting cellular immune responses and activation adaptive and innate immune responses through induction of plasmacytoid DC and B cells (29). It could also significantly enhance the number and survival of CD8^+^ T cells (30). CpG-ODNs could reinforce humoral and cellular immunity. A study showed that addition of CpG-ODNs as an adjuvant to the formulation of the HIV-1 Tat-based protein vaccines could enhance humoral and cellular immunity (31). Another study also indicated that a pcDNA3.1-tat DNA vaccine containing CpG-ODNs caused increased humoral immunity as well as lymphocyte proliferation and IFN-γ cytokine release in the mice model (10). Results of another study suggest that the DNA vaccine adjuvanted with CpG-ODNs induced specific IgG_1_, IgG_2a_, IFN-γ, and CTL responses remarkably. By contrast, the very same DNA vaccine without adjuvant was unable to elicit significant immune responses (32). Given all studies mentioned above, we sought to examine if CpG-ODNs adjuvant could improve the immunogenicity of the HIV-1 polyepitope DNA vaccine candidate. 

As mentioned before, induction of humoral responses and production of neutralizing antibodies, in particular in portal routes of HIV-1 (mucosal and systemic routes), is very important and has to be considered in designing prophylactic vaccines (2, 4, 5). Specific sera antibodies (particularly against gp41 and gp120) have been very efficient in prevention of HIV-1 infection (2, 4, 5). Our results showed that pcDNA3.1-HIV-1 polyepitope adjuvanted with CpG-ODNs could significantly increase IgG_1_ and IgG_2a_ antibodies in the immunized animals compared to the controls (Figures 2a and 2b). 

Cellular immune responses are the next important factor in controlling viral load and prevention of disease progression to AIDS, which should also be considered in a therapeutic vaccine’s design against HIV-1 (2, 4, 5). In this regard, we evaluated the induction of lymphocyte proliferative responses, cytokine IFN-γ production, and cytotoxicity activity of CTL as criteria for assessment of the candidate vaccines’ potencies in the elicitation of cellular immune responses (9, 17). This finding revealed that the HIV-1 polyepitope DNA based candidate vaccine could significantly strengthen lymphocyte proliferative responses, cytokine secretion, and cytotoxicity activity of CTLs in comparison with the control groups (Figures 3-5). 

## Conclusion

Taken together, the results of our study revealed that the HIV-1 polyepitope DNA candidate vaccine formulated with CpG-ODNs could significantly elicit immune responses in the vaccinated animals. It seems that the combination of some effective approaches in one immunization regime (using polytopic DNA vaccine along with applying human-compatible CpG-ODNs adjuvant and selection of a suitable route for vaccine administration) could boost the vaccine efficacy and activate simultaneously both arms of the immune system in immunized mice. Although this candidate vaccine still needs more investigation for its immunogenicity, efficacy profile, and safety, and further studies should be held to evaluate its potency, the findings of the present study show the efficacy of this HIV-1 polyepitopic candidate vaccine adjuvanted with CpG-ODNs and this approach could be considered a new approach in HIV-1 vaccine design.

## References

[1] Girard MP, Plotkin SA (2012). HIV vaccine development at the turn of the 21st century. Curr Opin HIV AIDS.

[2] Habibzadeh N, Bolhassani A, Vahabpour R, Sadat SM (2015). How can improve DNA vaccine modalities as a therapeutic approach against HIV infections?. J AIDS Clin Res.

[3] Barouch DH (2008). Challenges in the development of an HIV-1 vaccine. Nature.

[4] Lavanya J, Saxena S, Jais M, Dutta R (2013). DNA Vaccines-A Review. Jeevanu Times.

[5] Liu M (2003). DNA vaccines: A review. J Inter Med.

[6] Tan L, Zhang Y, Liu F, Yuan Y, Zhan Y, Sun Y (2016). Infectious bronchitis virus poly-epitope-based vaccine protects chickens from acute infection. Vaccine.

[7] Jazaeri EO, Mahdavi A, Abdoli A (2017). Formulation of chitosan with the polyepitope HIV-1 protein candidate vaccine efficiently boosts cellular immune responses in mice. Pathog Dis.

[8] Sun J, Hou J, Li D, Liu Y, Hu N, Hao Y (2013). Enhancement of HIV-1 DNA vaccine immunogenicity by BCG-PSN, a novel adjuvant. Vaccine.

[9] Jafarpour N, Memarnejadian A, Aghasadeghi MR, Kohram F, Aghababa H, Khoramabadi N (2014). Clustered epitopes within a new poly-epitopic HIV-1 DNA vaccine shows immunogenicity in BALB/c mice. Mol Biol Rep.

[10] Panahi Z, Abdoli A, Mosayebi G, Mahdavi M, Bahrami F (2018). Subcutaneous administration CpG-ODNs acts as a potent adjuvant for an HIV-1-tat-based vaccine candidate to elicit cellular immunity in BALB/c mice. Biotechnol Lett.

[11] Sambrook J, Fritsch EF, Maniatis T (1989). Molecular cloning.

[12] Kopycinski J, Cheeseman H, Ashraf A, Gill D, Hayes P, Hannaman D (2012). A DNA-based candidate HIV vaccine delivered via in vivo electroporation induces CD4 responses toward the α4β7-binding V2 loop of HIV gp120 in healthy volunteers. Clin Vaccine Immunol.

[13] Cafaro A, Tripiciano A, Sgadari C, Bellino S, Picconi O, Longo O (2015). Development of a novel AIDS vaccine: The HIV-1 transactivator of transcription protein vaccine. Expert Opin Biol Ther.

[14] Cicala C, Nawaz F, Jelicic K, Arthos J, S Fauci A (2016). HIV-1 gp120: A target for therapeutics and vaccine design. Curr Drug Targets.

[15] Yang Y, Zhu Q, Sun W, Guo J, Ning X, Li Q (2017). A recombinant multi-epitope protein MEP1 elicits efficient long-term immune responses against HIV-1 infection. Hum Vaccin Immunother.

[16] Reguzova A, Antonets D, Karpenko L, Ilyichev A, Maksyutov R, Bazhan S (2015). Design and evaluation of optimized artificial HIV-1 poly-T cell-epitope immunogens. PLoS one.

[17] Mahdavi M, Ebtekar M, Azadmanesh K, Mahboudi F, Khorramkhorshid H, Rahbarizadeh F (2010). HIV-1 Gag p24-Nef fusion peptide induces cellular and humoral immune response in a mouse model. Acta virologica.

[18] Ahmed T, Borthwick NJ, Gilmour J, Hayes P, Dorrell L, Hanke T (2016). Control of HIV-1 replication in vitro by vaccine-induced human CD8+ T cells through conserved subdominant Pol epitopes. Vaccine.

[19] Alipour S, Mahdavi A (2020). Boosting Tat DNA vaccine with Tat protein stimulates strong cellular and humoral immune responses in mice. Biotechnol Lett.

[20] Paul S, Planque SA, Nishiyama Y, Hanson CV (2009). A covalent HIV vaccine: is there hope for the future?. Future Virol.

[21] Falahati Z, Mahdavi A, Hassani L (2020). Physicochemical studies on the structural stability of the HIV-1 vaccine candidate recombinant Tat protein. Int J Biol Macromol.

[22] Kadkhodayan S, Jafarzade BS, Sadat SM, Motevalli F, Agi E, Bolhassani A (2017). Combination of cell penetrating peptides and heterologous DNA prime/protein boost strategy enhances immune responses against HIV-1 Nef antigen in BALB/c mouse model. Immunol Lett.

[23] Arabi S, Aghasadeghi MR, Memarnejadian A, Kohram F, Aghababa H, Khoramabadi N (2014). Cloning, expression and purification of a novel multi-epitopic HIV-1 vaccine candidate. A preliminary study on immunoreactivity..

[24] Agrawal S, Kandimalla ER (2002). Medicinal chemistry and therapeutic potential of CpG DNA. Trends Mol Med.

[25] Bode C, Zhao G, Steinhagen F, Kinjo T, Klinman DM (2011). CpG DNA as a vaccine adjuvant. Expert Rev Vaccines.

[26] Marciani DJ (2003). Vaccine adjuvants: Role and mechanisms of action in vaccine immunogenicity. Drug discovery today.

[27] Mutwiri G, Babiuk LA (2009). Approaches to enhancing immune responses stimulated by CpG oligodeoxynucleotides. Adv Drug Deliv Rev.

[28] Rees DC, Hartley MG, Green M, Lukaszewski RA, Griffin KF, Atkins HS (2013). The ability of CpG oligonucleotides to protect mice against Francisella tularensis live vaccine strain but not fully virulent F tularensis subspecies holarctica is reflected in cell-based assays. Microb Pathog.

[29] Caputo A, Gavioli R, Bellino S, Longo O, Tripiciano A, Francavilla V (2009). HIV-1 Tat-based vaccines: an overview and perspectives in the field of HIV/AIDS vaccine development. Int Rev Immunol.

[30] Ballas ZK, Rasmussen WL, Krieg AM (1996). Induction of NK activity in murine and human cells by CpG motifs in oligodeoxynucleotides and bacterial DNA. J Immunol.

[31] Alipour S, Mahdavi A, Abdoli A (2017). The effects of CpG-ODNs and Chitosan adjuvants on the elicitation of immune responses induced by the HIV-1-Tat based candidate vaccines in mice. Pathog Dis.

[32] Kojima Y, Xin K-Q, Ooki T, Hamajima K, Oikawa T, Shinoda K (2002). Adjuvant effect of multi-CpG motifs on an HIV-1 DNA vaccine. Vaccine.

